# Development and Internal Validation of the Palliative Metabolic Risk Score (PMRS) for Predicting Critical Outcome in Palliative Inpatients

**DOI:** 10.3390/healthcare14081041

**Published:** 2026-04-15

**Authors:** Muhammet Fatih Şahin, Ali Erol

**Affiliations:** 1 Department of Internal Medicine, Kestel State Hospital, 16100 Bursa, Türkiye; 2 Department of Internal Medicine, Bursa Yüksek Ihtisas Training and Research Hospital, 16310 Bursa, Türkiye

**Keywords:** palliative care, in-hospital critical outcome, malignancy, hyperglycemia, oxygen requirement

## Abstract

**Background/Objectives:** In-hospital critical outcome among palliative inpatients remains high, often driven by acute physiological instability rather than chronic comorbidities. Although diabetes mellitus (DM) is common in this population, its independent impact on critical outcome is unclear. This study aimed to determine whether acute metabolic and inflammatory markers—specifically glucose, C-reactive protein (CRP), albumin, and oxygen requirement—better predict short-term outcomes, defined as in-hospital critical outcome or ICU transfer during the same hospitalization period, than DM status alone. **Methods:** This retrospective study included 200 palliative inpatients admitted to the Internal Medicine Clinic of Kestel State Hospital, Bursa, Turkey, between January 2024 and January 2025. Demographic, clinical, and laboratory data were obtained from electronic records. The primary outcome was in-hospital critical outcome or ICU transfer (“critical outcome”). Logistic regression and receiver-operating characteristic (ROC) analyses identified independent predictors. The study was approved by the Bursa Yüksek İhtisas Training and Research Hospital Ethics Committee (ethics approval: protocol code 2024-TBEK 2025/05-12). **Results:** The mean age was 77.7 ± 12.3 years, and 47% were male. DM was present in 30.5% but did not independently predict critical outcome (*p* = 0.904). In contrast, oxygen requirement (OR = 4.08, *p* = 0.002), mean glucose (OR = 1.01, *p* = 0.001), and cancer (OR = 3.28, *p* = 0.016) were significant predictors. ROC analysis identified CRP > 64.1 mg/L and albumin < 25 g/L as optimal thresholds, and these two markers formed the basis of the low-, intermediate-, and high-risk stratification, with critical-outcome rates of 39.0%, 45.1%, and 85.4% (*p* < 0.001). **Conclusions:** Acute metabolic and inflammatory disturbances—particularly hyperglycemia, elevated CRP, hypoalbuminemia, and oxygen requirement—are stronger prognostic indicators than DM. A simple bedside model incorporating these parameters may improve prognostic accuracy and communication in palliative care.

## 1. Introduction

Palliative care focuses on improving quality of life and alleviating suffering in patients with advanced and life-limiting illnesses. Despite its supportive intent, in-hospital critical outcome remains high among palliative patients, highlighting the importance of identifying reliable prognostic indicators to guide treatment decisions, resource allocation, and communication with patients and families. Recent landmark studies in Palliative Medicine have emphasized the need for objective and reproducible prognostic tools to improve the accuracy of survival prediction in end-of-life care [1,2].

Diabetes mellitus (DM) is one of the most common comorbidities among palliative inpatients, affecting nearly one-third of hospitalized patients [3,4]. It contributes to increased infection risk, delayed wound healing, and metabolic instability, which may worsen short-term prognosis. However, current evidence suggests that diabetes mellitus itself is not an independent predictor of short-term adverse outcomes in this setting, whereas acute metabolic and inflammatory disturbances appear to have greater prognostic relevance [5,6]. Emerging evidence suggests that acute physiological disturbances—particularly hyperglycemia, systemic inflammation, and oxygen requirement—play a stronger role in determining short-term outcomes than chronic comorbidities in the terminal phase of illness [5,7].

The landmark NICE-SUGAR trial demonstrated that intensive glycemic control increased critical outcome by elevating the risk of hypoglycemia, underscoring the need for individualized and moderate glucose targets in critically ill patients [8]. In palliative and advanced disease populations, moderate glycemic control (180–250 mg/dL) has been shown to minimize metabolic stress without worsening outcomes.

Previous studies have demonstrated that systemic inflammation, particularly reflected by elevated C-reactive protein (CRP) and decreased albumin, independently predicts short-term survival in advanced cancer patients [9]. The CRP/albumin ratio (CAR), which integrates both inflammation and nutritional status, has been validated as a robust prognostic marker in terminally ill patients [10,11,12].

Importantly, while procalcitonin (PCT) is a highly infection-specific biomarker, it has limited prognostic value in chronic or multifactorial inflammatory states. In contrast, CRP reflects a broader systemic inflammatory and metabolic decline, providing a more comprehensive and practical indicator of prognosis in end-stage conditions—especially in resource-limited palliative settings [13].

Despite this growing body of evidence, studies simultaneously evaluating hyperglycemia, CRP, albumin, and oxygen requirement as integrated prognostic factors in palliative inpatients are scarce. Furthermore, most available data come from tertiary centers, while real-world data from secondary hospitals—where a large proportion of palliative care is actually delivered—are limited [14,15]. Unlike previous prognostic indices such as the Palliative Prognostic Index (PPI), the Palliative Metabolic Risk Score (PMRS) focuses exclusively on objective laboratory and physiological parameters applicable to real-world secondary hospitals [1].

Therefore, this study aimed to evaluate the relationship between diabetes mellitus and in-hospital critical outcome among palliative care patients and to identify independent predictors of poor outcomes based on routinely measurable biochemical and clinical parameters. By integrating easily measurable metabolic and inflammatory parameters, this study develops a simple, objective, and bedside-applicable prognostic model (PMRS) to improve risk stratification in real-world palliative care settings.

## 2. Materials and Methods

### 2.1. Study Design and Ethical Approval

This retrospective, observational study was designed to evaluate critical-outcome predictors among palliative inpatients with and without diabetes mellitus (DM). Ethical approval was obtained from the Medical Sciences Ethics Committee of the University of Health Sciences Bursa Yüksek Ihtisas Training and Research Hospital (Approval No: 2024-TBEK 2025/05-12). Because this was a retrospective study using fully anonymized data, the ethics committee waived the requirement for informed consent. All procedures were performed in accordance with relevant guidelines and regulations, including the Declaration of Helsinki.

### 2.2. Study Population

A total of 200 adult patients (≥18 years) who were hospitalized in the palliative care unit between 1 January 2024 and 1 January 2025 were retrospectively reviewed. The study included patients admitted to the palliative care unit for active inpatient medical management. Patients receiving hospice-only comfort care without active medical treatment were not included. Admission to the palliative care unit was determined according to routine institutional clinical practice, based on advanced chronic illness, functional impairment, symptom burden, and supportive care needs, rather than on predefined metabolic or inflammatory laboratory parameters. To ensure the independence of observations and avoid duplication of patient data, repeat admissions were excluded; only the first hospitalization during the study period was analyzed for each patient. Patients transferred from other institutions or with incomplete records were excluded.

For each patient, demographic characteristics, comorbidities, primary diagnosis, medication use, laboratory parameters and hospitalization outcomes were obtained from the hospital’s electronic medical records. Mean glucose was calculated as the arithmetic mean of available in-hospital blood glucose measurements recorded during routine clinical care for each patient; because of the retrospective design, the timing and frequency of these measurements were not fully standardized across patients. The analyzed variables included demographic characteristics (age and sex), diabetes status, primary diagnosis (including cancer), oxygen requirement, feeding route, laboratory parameters (glucose, CRP, and albumin), medication use, ICU transfer, in-hospital critical outcome, and length of hospital stay. The primary exposure variable was the presence of diabetes mellitus, defined by physician-documented diagnosis or the use of antidiabetic medication. Feeding route (oral, nasogastric [NG] tube, or percutaneous endoscopic gastrostomy [PEG]), oxygen requirement, and cancer diagnosis were recorded as relevant clinical parameters.

### 2.3. Definitions and Outcomes

The primary outcome was in-hospital critical outcome. In this study, the short-term critical outcome was defined as a composite endpoint including in-hospital death or transfer to the intensive care unit (ICU) during the index hospitalization. A critical outcome was defined as either in-hospital death or transfer to the ICU. This composite definition was used to capture major clinical deterioration during hospitalization; however, ICU transfer may also reflect institutional practices, resource availability, physician judgment, and goals of care in addition to biological severity. Secondary outcomes included hospital length of stay and feeding route.

CRP, albumin, and glucose levels were analyzed both as continuous and categorized variables, using ROC-derived cut-offs. All data were anonymized prior to analysis.

### 2.4. Statistical Analysis

Statistical analyses were performed using IBM SPSS Statistics version 22.0 (IBM Corp., Armonk, NY, USA). Continuous variables were expressed as mean ± standard deviation (SD) or median (interquartile range), while categorical variables were expressed as frequencies and percentages. Normality was assessed by Kolmogorov–Smirnov and Shapiro–Wilk tests. Comparisons between DM and non-DM groups were conducted using Student’s *t*-test or the Mann–Whitney U test for continuous data and the Chi-square test for categorical data. Correlations between continuous variables were analyzed using Pearson or Spearman coefficients, as appropriate.

Independent predictors of critical outcome were identified using multivariate logistic regression including variables that were significant in univariate analyses. Variables with *p* < 0.10 in univariate logistic regression were entered into the multivariable logistic regression model. Multicollinearity was assessed using variance inflation factor (VIF) and tolerance statistics before final model construction. CRP and albumin were analyzed as separate variables rather than combined into the C-reactive protein-to-albumin ratio (CAR), in order to preserve clinical interpretability and avoid redundancy from including a derived composite variable based on two already measured predictors. For development of the PMRS, independent predictors identified in the multivariable logistic regression model were converted into an integer-based bedside score according to the relative magnitude of their β coefficients, and the performance of the simplified score was evaluated by ROC analysis, calibration assessment, and bootstrap internal validation. Model calibration and discrimination were evaluated using the Hosmer–Lemeshow test and receiver-operating characteristic (ROC) analysis, respectively. Multiple linear regression was applied to determine predictors of hospital length of stay. Statistical significance was set at *p* < 0.05 (two-tailed). A post hoc power analysis based on the area under the ROC curve (area under the curve (AUC) = 0.772) was conducted to verify the model’s discriminative strength. With a total sample size of 200 and α = 0.05, the achieved statistical power to detect AUC > 0.5 was greater than 99.9% across plausible event rates (30–70%). The events-per-variable (EPV) ratio was calculated to assess the risk of overfitting. With 66 events and five predictors in the final model, the EPV was approximately 13.2, which is above the commonly recommended threshold of 10 [16].

## 3. Results

### 3.1. Study Population and Baseline Characteristics

A total of 200 palliative inpatients were included in the final analysis. The mean age was 77.7 ± 12.3 years, and 47.0% were male. Diabetes mellitus (DM) was present in 61 patients (30.5%), and cancer constituted 22.0% of the cohort. When patients were grouped according to critical-outcome status, mean glucose and CRP levels were significantly higher in those with critical outcome than in those without critical outcome (149.9 ± 66.2 vs 116.8 ± 49.7 mg/dL, *p* < 0.001; and 66.2 ± 42.2 vs 47.9 ± 41.6 mg/L, *p* = 0.002, respectively). Cancer as the primary diagnosis was also more frequent among patients with critical outcome (75.0% vs 25.0%, *p* = 0.003). No significant differences were observed between the groups in age, sex, DM status, or albumin level (Table 1).

### 3.2. Clinical Outcomes

In-hospital critical outcome occurred in 66 patients (33%), with no significant difference between DM and non-DM groups (34.4% vs 32.4%, *p* = 0.904). When outcomes were analyzed by primary diagnosis, critical outcome was markedly higher among cancer patients compared with non-cancer patients (75% vs 49%, *p* = 0.004). In contrast, the presence of diabetes was not associated with a statistically significant increase in critical outcome (*p* = 0.223).

Multivariate logistic regression identified oxygen requirement (OR = 4.08, 95% CI 1.65–10.14, *p* = 0.002), higher mean glucose levels (OR = 1.01, 95% CI 1.01–1.02, *p* = 0.001), and cancer diagnosis (OR = 3.28, 95% CI 1.25–8.59, *p* = 0.016) as independent predictors of critical outcome (Table 2). Although CRP > 64.1 mg/L did not retain statistical significance in the multivariable model, it was included in the PMRS due to its strong univariate association with critical outcomes and its established clinical relevance as an inflammatory severity marker.

### 3.3. Predictors of Critical Outcome

Receiver-operating characteristic (ROC) analyses identified the optimal discriminative thresholds as 64.1 mg/L for CRP (AUC = 0.63) and 25 g/L for albumin (AUC = 0.62) for predicting critical outcome.

**Figure 1:**
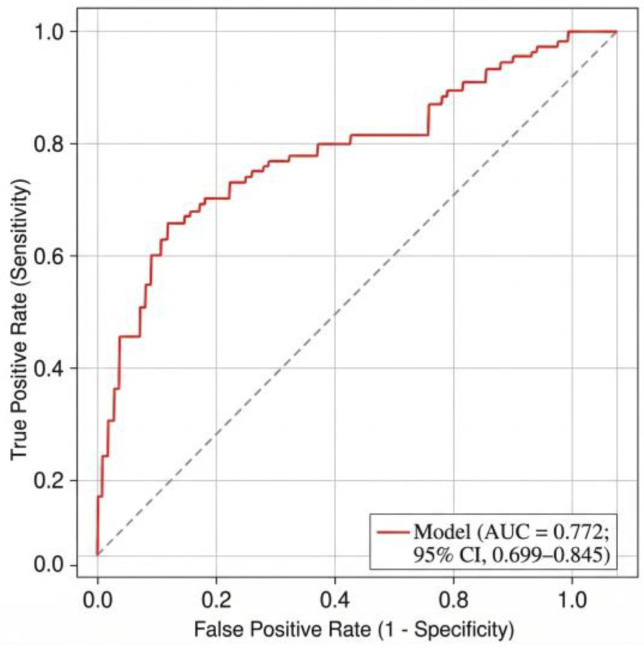
Receiver Operating Characteristic (ROC) curve for the multivariable model predicting critical outcome. **Note**: Figure 1. ROC curve for the multivariable logistic regression model predicting critical outcome (AUC = 0.772; 95% CI 0.699–0.845). The dotted diagonal line represents the line of no discrimination (AUC = 0.5).

Based on these cut-offs, patients were stratified into three risk categories—low, intermediate, and high—corresponding to critical-outcome rates of 39.0%, 45.1%, and 85.4%, respectively (*p* < 0.001). The model demonstrated clear stepwise separation between categories (Table 3, Figure 1).

Feeding route analysis revealed that critical outcome differed among groups: 56.0% for oral feeding, 72.4% for nasogastric (NG) feeding, and 37.8% for percutaneous endoscopic gastrostomy (PEG) (*p* = 0.018) (Table 4).

Multiple linear regression identified pressure ulcer as the sole independent factor prolonging hospital stay by approximately 12 days (β = +12.34, *p* = 0.002), while in-hospital death tended to shorten stay (β = −7.06, *p* = 0.078) (Supplementary Table S1).

Univariate associations between individual comorbidities and critical outcome are presented in Supplementary Table S2. In addition, subgroup analyses in diabetic patients are provided in Supplementary Table S3, consistent with limited statistical power.

Overall, these findings indicate that acute metabolic and physiological factors—oxygen requirement, hyperglycemia, systemic inflammation, and feeding route—predominantly determine clinical outcomes in palliative inpatients, rather than chronic comorbidities such as diabetes alone.

### 3.4. Model Development and Risk Scoring

To improve clinical interpretability, we developed a simplified “Palliative Metabolic Risk Score (PMRS)” using five independent predictors identified in the multivariable model (oxygen requirement, CRP, albumin, glucose, and malignancy). Each variable was assigned an integer score proportional to its β coefficient (Table 5).

**Figure 2:**
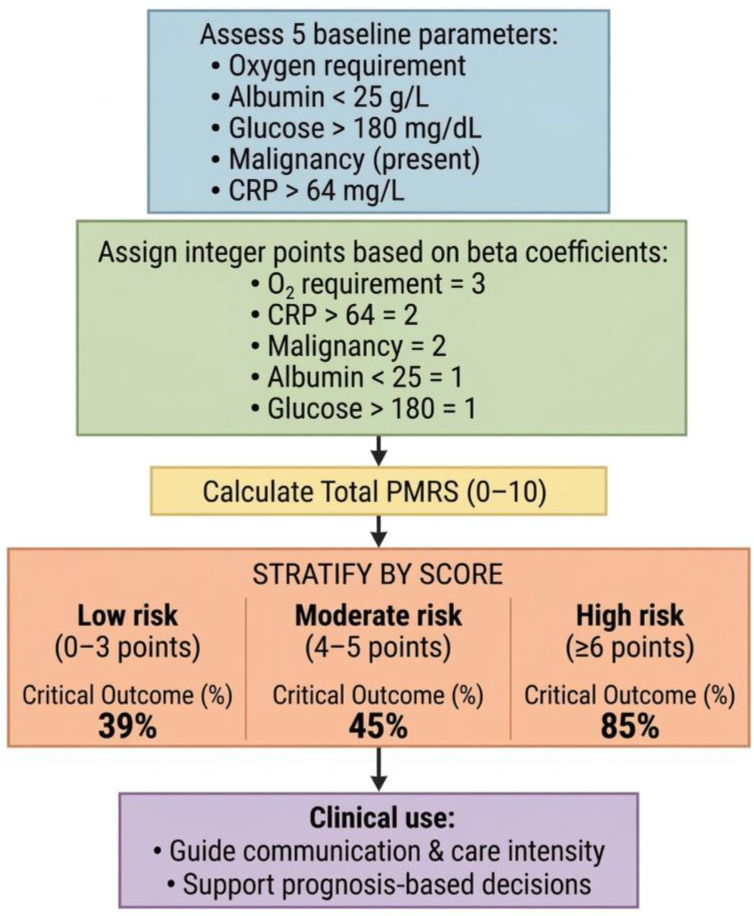
Clinical flowchart for the bedside application and risk stratification of the Palliative Metabolic Risk Score (PMRS).

The total score ranged from 0 to 10, and higher scores indicated greater critical outcome risk. The stepwise development and bedside application of the PMRS are illustrated in Figure 2, showing the five input variables, corresponding point allocation, total score calculation, and the risk stratification pathway.

### 3.5. Model Performance and Calibration

**Figure 3:**
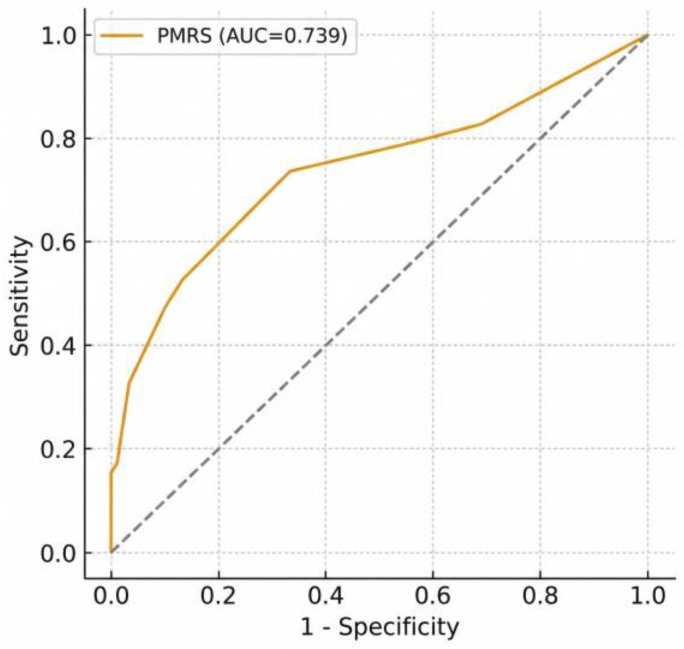
Receiver operating characteristic (ROC) curve analysis of the Palliative Metabolic Risk Score (PMRS). **Note:** ROC curve analysis of the simplified PMRS (AUC = 0.739), demonstrating discriminative performance for clinical use. The dotted diagonal line represents the line of no discrimination (AUC = 0.5).

**Figure 4:**
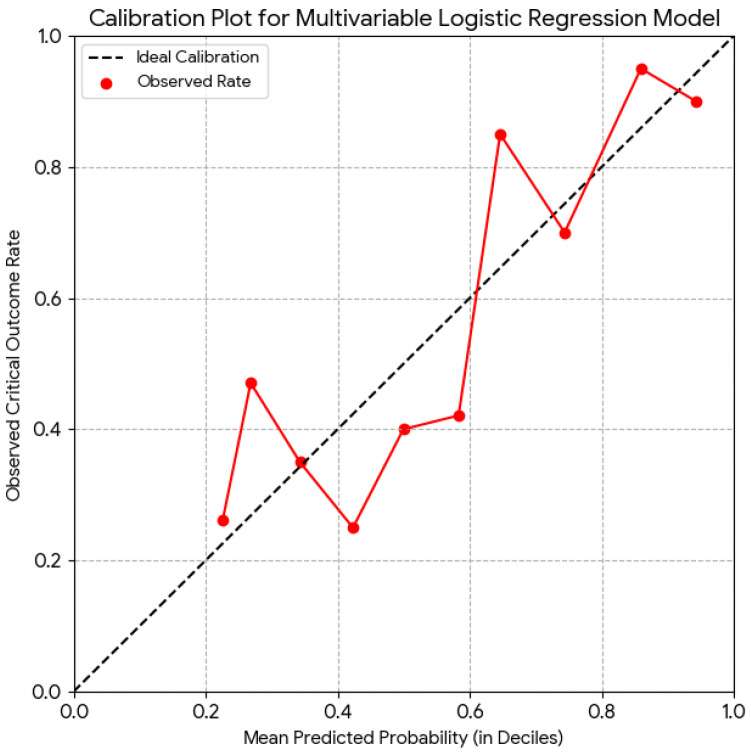
Calibration plot comparing predicted and observed probabilities for the multivariable logistic regression model. **Note:** The dashed black line represents the Ideal Calibration (perfect calibration), where predicted probabilities perfectly match observed outcome rates. The red dots, connected by a solid red line, represent the Observed Rate of the critical outcome calculated for ten deciles of predicted probability. Although the curve broadly follows the diagonal line, notable deviations are visible, particularly in the mid-to-high probability range (0.4 to 0.7), suggesting areas where the model may over-predict (below the line) or under-predict (above the line) risk. The plot indicates moderate but imperfect model calibration and may require further refinement to improve performance.

The logistic regression model achieved good discrimination for predicting in-hospital critical outcome or ICU transfer (AUC = 0.772, 95% CI 0.699–0.845), while the simplified PMRS retained comparable performance (AUC = 0.739; sensitivity = 0.74; specificity = 0.67). The receiver operating characteristic (ROC) curve is shown in Figure 3, confirming good discrimination, whereas the calibration plot (Figure 4) demonstrated close agreement between predicted and observed outcome probabilities across deciles of risk, supporting good model fit and reliability. The overall Brier score of 0.192 indicated low prediction error and good model calibration, consistent with the Hosmer–Lemeshow test (*p* = 0.64).

### 3.6. Internal Validation

To assess the internal stability of the PMRS model, bootstrap resampling with 1000 iterations was performed. The bias-corrected odds ratios of the five predictors remained consistent with the original estimates (Supplementary Table S4). Oxygen requirement, mean glucose level, albumin < 25 g/L, and primary diagnosis of cancer retained statistical significance in the resampled datasets. The CRP > 64.1 mg/L variable showed a stable but borderline effect. The narrow 95% bootstrap confidence intervals confirmed minimal estimation bias and high internal reproducibility of the PMRS predictors. Taken together, these findings confirm that the PMRS demonstrates stable internal performance with acceptable discrimination and calibration for bedside clinical use. However, although bootstrap resampling supports the internal stability of the multivariable model, a separate bootstrap-based stability analysis of the ROC-derived cut-off values themselves was not specifically performed. Therefore, the selected thresholds should be interpreted cautiously and require confirmation in independent external cohorts. Additional exploratory multivariable analyses examining the interaction between diabetes mellitus and glucose levels are presented in Supplementary Table S5.

## 4. Discussion

This study demonstrated that critical outcome and other adverse outcomes among palliative inpatients are driven more strongly by acute physiological deterioration—particularly hyperglycemia and oxygen requirement—rather than by chronic comorbidities such as diabetes mellitus [3,17]. Although diabetes was present in approximately one-third of the cohort, it was not an independent predictor of critical outcome. Additional exploratory analyses including a diabetes × glucose interaction suggested that the prognostic impact of hyperglycemia may differ according to diabetes status, although this interaction became borderline after centering the glucose variable. These findings support the interpretation that part of the prognostic effect attributed to diabetes may be mediated or modified by acute hyperglycemia. However, this finding should be interpreted cautiously, because glucose is biologically and clinically linked to diabetes status and may lie on the causal pathway between diabetes and adverse outcomes. Therefore, adjustment for glucose may have partially attenuated the apparent independent prognostic effect of diabetes through overadjustment, suggesting not that diabetes is irrelevant to prognosis but that part of its effect may be mediated through acute hyperglycemia. In contrast, mean glucose level and oxygen requirement emerged as the strongest prognostic indicators, suggesting that in the palliative phase, acute metabolic and respiratory instability outweighs long-term diagnoses in determining patient trajectories [4].

Our findings parallel the results of the NICE-SUGAR study, which showed that intensive glycemic control increased critical outcome by raising the risk of hypoglycemia [8]. In palliative care, where nutritional status, inflammation, and systemic stress vary considerably, a liberal glucose control strategy appears physiologically more appropriate [7,18]. The observed association between admission hyperglycemia and critical outcome, independent of diabetes status, is consistent with previous studies [5,6]. These data support the implementation of individualized glycemic targets rather than uniform insulin protocols [19].

Hypoglycemia, on the other hand, represents a serious source of morbidity and distress in terminally ill patients [20]. Therefore, moderate glycemic targets (180–250 mg/dL) are recommended to minimize metabolic stress while avoiding hypoglycemia in end-stage patients [21,22,23].

Overall, this study indicates that it is not the diagnosis of diabetes itself but uncontrolled hyperglycemia that is linked to critical outcome—highlighting a transition from chronic disease management to acute metabolic stabilization in palliative care.

Elevated CRP levels were consistently associated with critical outcome. The optimal CRP threshold identified in this study (64.1 mg/L) aligns with prior findings in patients with advanced cancer or end-stage organ failure, in whom similar cut-offs have prognostic value [11,12]. Similar observations were reported by Hui et al., who demonstrated that inflammatory markers such as CRP and albumin are independent predictors of short-term survival in advanced cancer patients receiving palliative care [9]. Systematic reviews have highlighted them as a robust marker of systemic inflammation and catabolic stress [24].

When combined with albumin, the CAR further enhances prognostic accuracy, reflecting both inflammation and malnutrition [10,25]. Likewise, low albumin levels correlate with protein-energy loss, reduced physiological reserve, and shorter survival [26,27].

This superiority is reinforced by the finding that CRP reached statistical significance, whereas PCT, a biomarker typically regarded as more specific for infection, did not (*p* = 0.2186). This suggests that in palliative settings, where cost-effectiveness and rapid decision-making are essential, CRP may serve as a more practical and sufficiently prognostic triage tool than PCT. Being a broader marker of systemic inflammation, CRP better reflects overall physiological decline rather than infection alone [13,28].

The presence of malignancy independently increased critical outcome, reflecting the combined impact of systemic inflammation, catabolism, and treatment-related metabolic vulnerability in cancer patients [29,30]. Previous research has similarly reported that the coexistence of elevated CRP, hypoalbuminemia, and glucose imbalance correlates with poor prognosis in malignancy [31,32,33]. Conversely, chronic non-malignant conditions such as COPD and stroke were inversely associated with critical outcome—a likely manifestation of selection bias. These patients are often admitted for symptom control rather than acute deterioration, which explains their relatively prolonged survival [34,35]. Collectively, these findings confirm that the model accurately captures acute decompensation arising from high-risk clinical conditions such as malignancy.

Our results also revealed that patients fed via nasogastric (NG) tubes exhibited significantly higher critical outcome than those fed orally or via percutaneous endoscopic gastrostomy (PEG). Although PEG-fed patients typically have worse baseline conditions, their lower critical-outcome rates may reflect the method’s greater safety, lower aspiration risk, and better long-term maintenance. NG feeding, by contrast, is often used in acutely decompensated or terminal patients, where aspiration, secretion management, and tube obstruction contribute to poorer outcomes. Therefore, the elevated critical outcome among NG-fed patients likely reflects the combination of acute physiological instability and care-related complications rather than the feeding route itself.

The presence of pressure ulcers prolonged hospital stay by an average of 12 days, consistent with previous studies showing that such ulcers extend hospitalization but do not increase critical outcome [36,37].

Our multivariate model stratified patients into three prognostic categories, with critical-outcome rates rising from 39% to 45% and 85%. This distribution mirrors the accuracy of simplified models combining cancer diagnosis, CRP, albumin, glucose, and oxygen requirement [14,38,39].

Oxygen requirement itself increased critical-outcome risk nearly threefold, consistent with prior findings indicating that oxygen dependence reflects acute decompensation rather than chronic failure [40,41]. The model integrating these parameters provides a practical, bedside-applicable risk stratification system that captures both physiological instability and care dependency [15,42]. This framework enables clinicians and families to make more realistic prognostic assessments and facilitates more efficient allocation of healthcare resources [4,43]. These findings are in line with the broader prognostic research framework emphasizing the integration of clinical and laboratory indicators to improve end-of-life prediction accuracy [44].

Several established prognostic indices, such as the PPI and Palliative Prognostic Score (PaP), have been widely used to estimate short-term survival in palliative populations [2]. The most widely validated among these is the Palliative PPI, developed and externally validated as a simple bedside tool integrating clinical and functional variables [1]. However, these models primarily rely on subjective clinical assessments (e.g., performance status, oral intake, and clinician-estimated survival) and require trained evaluators, which may limit their reproducibility and applicability in busy or resource-limited settings [25]. In the present study, several of these core variables were not systematically available in the retrospective dataset, precluding direct calculation and head-to-head performance comparison with the PPI or PaP. Therefore, any comparison between the PMRS and these established tools should be interpreted as conceptual rather than empirical. However, the PMRS offers a more objective, laboratory-based alternative applicable in data-limited or emergency settings, where functional or symptom-based assessments may not be feasible. The PMRS is not intended to replace established prognostic indices, but rather to provide a simple adjunctive bedside tool based on routinely available clinical and laboratory variables. Its potential value lies in supporting early risk stratification, closer monitoring, multidisciplinary reassessment, and clinically informed communication within the context of comprehensive patient evaluation. Importantly, the PMRS should be used to support communication and shared decision-making, rather than as a directive instrument to restrict care, and its results should always be interpreted in light of overall clinical judgment, patient values, and individualized goals of care.

In contrast, the PMRS developed in this study is based entirely on objective, routinely available biochemical and clinical parameters—glucose, CRP, albumin, oxygen requirement, and malignancy. By excluding subjective inputs, the PMRS offers a simplified, low-cost, and bedside-applicable alternative for risk stratification that can be calculated automatically from laboratory and monitoring data. Similar objective laboratory-based prognostic approaches have been successfully applied in other chronic disease settings, further supporting the utility of measurable physiological parameters in risk stratification. For example, the correlation between renal function stages and high-frequency hearing loss in chronic kidney disease patients [45] illustrates how quantifiable biological markers can reflect systemic deterioration across organ systems. These observations reinforce that objective biochemical parameters provide a reproducible and clinically practical framework for outcome prediction in end-stage conditions.

Furthermore, while tools such as PPI and PaP are primarily validated in cancer populations, the PMRS was developed in a real-world palliative inpatient cohort including both malignant and non-malignant conditions, with non-malignant conditions predominating [14]. This enhances its potential generalizability to broader palliative inpatient populations.

In addition to identifying individual predictors, we developed a simplified bedside scoring system, the PMRS, to facilitate early risk stratification. The score, derived from five independent predictors (oxygen requirement, CRP, albumin, glucose, and malignancy), demonstrated good discriminative capacity (AUC = 0.739) compared with the full logistic model (AUC = 0.772). This indicates that, despite minor loss of precision due to categorical simplification, the PMRS preserves clinically meaningful accuracy and may be more practical for real-world decision-making. Such parsimonious tools can support physicians in identifying high-risk patients and tailoring care intensity in palliative units. Beyond discrimination, the model also exhibited good calibration, as evidenced by a low Brier score (0.192) and visual agreement in the calibration plot, suggesting that predicted risks accurately reflected observed event rates across probability strata.

Despite its methodological strengths, this study has several limitations. Its retrospective and single-center design limits causal inference and generalizability. In addition, because of the retrospective single-center design, admission to the palliative care unit may have been influenced by clinician judgment and underlying disease severity, which could have affected critical-outcome risk independently of the metabolic or inflammatory markers evaluated. Because glucose measurements were obtained retrospectively from routine clinical practice rather than from predefined standardized time points, inter-patient differences in the timing and frequency of glucose assessment may have introduced variability into the mean glucose estimates. The CRP and albumin thresholds used in the PMRS were derived from ROC analyses within the present cohort and should therefore be considered internally derived exploratory cut-offs rather than universally established clinical thresholds. Their stability and generalizability should be confirmed in independent external populations. Furthermore, due to the retrospective nature of this study, DNR (do-not-resuscitate) status was not systematically documented in the electronic medical records in a structured format. Consequently, this variable could not be reliably analyzed or included in the prognostic models, which represents a limitation of the present study. The composite critical outcome included both in-hospital critical outcome and ICU transfer. Because ICU transfer decisions may also be influenced by institutional practices, resource availability, physician judgment, and goals of care, some heterogeneity in the outcome definition cannot be excluded. Although the PMRS showed acceptable discrimination and internal stability, the score should be regarded as exploratory and hypothesis-generating, since optimism-corrected discrimination metrics, calibration slope/intercept estimates, and decision curve analysis were not available, and external validation in an independent cohort has not yet been performed. Because data were extracted from electronic medical records, some laboratory and clinical variables may have been missing or inconsistently documented. Potential confounders such as acute infection status, infection focus, corticosteroid or other immunosuppressive therapy, nutritional interventions, intravenous fluid therapy, and detailed nutritional assessments were not consistently available in a structured form in the retrospective records and therefore could not be included in the adjusted analyses. As a result, residual confounding cannot be excluded, and some of these factors may have influenced the observed metabolic parameters. DM was evaluated as a binary variable, and detailed data on disease duration, treatment type, and prior long-term glycemic control were not consistently available in the retrospective records. Therefore, the absence of these diabetes-specific characteristics may have limited the ability to detect potential prognostic differences within the diabetic subgroup. Because this study was conducted in a single secondary-care hospital, the generalizability of the PMRS to tertiary referral centers or hospice-based palliative care settings remains uncertain. Differences in patient case-mix, resource availability, escalation practices, and care goals may influence both the score’s performance and its clinical applicability in other settings.

The sample sizes of the diabetic and cancer subgroups were relatively small, potentially reducing the statistical power to detect subgroup-specific associations. Although the PMRS demonstrated good internal discrimination and calibration (AUC = 0.739; Brier score = 0.192; Hosmer–Lemeshow *p* = 0.64) and was internally validated through 1000 bootstrap resamples, the model has not yet undergone external validation in independent cohorts. Therefore, its predictive performance should be regarded as internally derived and hypothesis-generating rather than confirmatory, warranting further multicenter validation to establish its generalizability.

Because the cohort reflects a real-world palliative population, certain unmeasured confounders—such as disease stage, symptom burden, nutritional status, or concurrent infections—may still have influenced outcomes. Future multicenter, prospective studies with external validation and standardized variable documentation are warranted to confirm the reproducibility, calibration stability, and broader clinical applicability of the PMRS.

## 5. Conclusions

This study suggests that in palliative inpatients, acute metabolic and inflammatory markers are more directly associated with clinical outcomes than chronic comorbidities alone. Glucose control, oxygen requirement, and CRP level are easily accessible and clinically meaningful prognostic indicators. Nevertheless, the role of diabetes should be interpreted cautiously, as part of its prognostic effect may be mediated through acute hyperglycemia.

Incorporating these parameters into routine monitoring could enhance prognostic accuracy, guide treatment decisions, and improve communication with patients and families. However, external validation of the PMRS in independent cohorts is essential before widespread clinical adoption.

## Figures and Tables

**Table 1:** Baseline characteristics according to critical outcome status.

Variable	Total (*n* = 200)	Critical Outcome: No (*n* = 90)	Critical Outcome: Yes (*n* = 110)	*p*-Value
Age (years), mean ± SD	77.7 ± 12.3	78.4 ± 11.7	77.1 ± 12.9	0.465
Male sex, *n* (%)	94 (47.0)	38 (40.4)	56 (59.6)	0.221
Diabetes mellitus, *n* (%)	61 (30.5)	23 (37.7)	38 (62.3)	0.170
Mean glucose (mg/dL), mean ± SD	133.9 ± 63.1	116.8 ± 49.7	149.9 ± 66.2	**<0.001**
CRP (mg/L), mean ± SD	58.0 ± 42.8	47.9 ± 41.6	66.2 ± 42.2	**0.002**
Albumin (g/L), mean ± SD	20.8 ± 11.0	22.4 ± 11.0	19.6 ± 10.9	0.078
Cancer as primary diagnosis, *n* (%)	44 (22.0)	11 (25.0)	33 (75.0)	**0.003**

**Note:** Continuous data are expressed as mean ± SD, categorical data as *n* (%); *p*-values obtained using Student’s *t*-test or Mann–Whitney U for continuous variables and Chi-square test for categorical variables. Significant *p*-values (*p* < 0.05) are marked as bold. **Abbreviations:** CRP, C-reactive protein.

**Table 2:** Univariate and multivariable logistic regression analyses for predictors of critical outcome.

Variable	Crude OR (95% CI)	*p*-Value	Adjusted OR (95% CI)	*p*-Value
Oxygen requirement	4.59 (2.12–9.95)	**<0.001**	4.08 (1.63–10.20)	**0.002**
Mean glucose (mg/dL)	1.01 (1.00–1.01)	**<0.001**	1.01 (1.00–1.02)	**0.001**
Primary diagnosis (Cancer)	3.08 (1.45–6.50)	**0.003**	3.28 (1.24–8.69)	**0.016**
CRP > 64.1 mg/L	2.67 (1.47–4.83)	**0.001**	1.98 (0.94–4.20)	0.071
Albumin < 25 g/L	1.85 (0.95–3.50)	0.070	2.21 (1.07–4.56)	**0.032**

**Note:** Model performance: AUC = 0.772; Hosmer–Lemeshow *p* = 0.64; Nagelkerke R^2^ = 0.38. CI, confidence interval; OR, odds ratio. Significant *p*-values (*p* < 0.05) are marked as bold.

**Table 3:** Critical-outcome rates according to risk categories.

PMRS Risk Category	Patients (*n*)	Critical Outcome (%)	95% CI
Low risk (0–3 points)	64	39.1	27.9–51.5
Intermediate risk (4–5 points)	66	45.5	33.7–57.8
High risk (≥6 points)	65	86.2	75.3–92.8

**Note:** Risk categories were based on composite model including oxygen requirement, mean glucose, cancer diagnosis, and CRP/albumin thresholds. Critical outcome was defined as in-hospital critical outcome or ICU transfer. Differences among categories were highly significant (*p* < 0.001).

**Table 4:** Critical outcome according to feeding route.

Feeding Route	Critical Outcome Rate (%)	*p*-Value
Oral feeding	75/134 (56.0)	
Nasogastric (NG)	21/29 (72.4)	**0.018**
PEG feeding	14/37 (37.8)	

**Note:** Significant *p*-values (*p* < 0.05) are marked as bold.

**Table 5:** Derivation and point allocation of the Palliative Metabolic Risk Score (PMRS).

Predictor Variable	Adjusted OR (95% CI)	β-Coefficient *	*p*-Value	Assigned Integer Points
Oxygen requirement	4.08 (1.63–10.20)	1.41	0.002	3
Primary diagnosis: cancer	3.28 (1.24–8.69)	1.19	0.016	2
CRP > 64.1 mg/L	1.98 (0.94–4.20)	0.68	0.071	2
Albumin < 25 g/L	2.21 (1.07–4.56)	0.79	0.032	1
Glucose > 180 mg/dL	1.01 (1.00–1.02) **	0.01 **	0.001	1

**Note:** β coefficients were derived from the final multivariable logistic regression model. Glucose was analyzed as a continuous variable in the regression model to preserve statistical power, where the OR of 1.01 represents the risk increase per 1 mg/dL. For bedside PMRS application, a clinical threshold of >180 mg/dL was adopted to align with international palliative care approaches to hyperglycemia. * Odds ratio expressed per 1 mg/dL increase in glucose. ** β-coefficient corresponding to glucose analyzed as a continuous variable.

## Data Availability

The datasets generated and/or analyzed during the current study are not publicly available due to institutional and ethical restrictions but can be made available from the corresponding author upon reasonable request. No AI systems were used for study design, data analysis, interpretation of results, figure or table generation, or scientific decision-making. All AI-assisted outputs were carefully reviewed and edited by the authors. The authors take full responsibility for the content and conclusions of this manuscript.

## Supplementary Materials

The following supporting information can be downloaded at https://www.mdpi.com/article/10.3390/healthcare14081041/s1, Table S1: Multiple linear regression for hospital length of stay; Table S2: Univariate association between comorbidities and critical outcome; Table S3: Subgroup analysis in diabetic patients; Table S4: Palliative Metabolic Risk Score (PMRS); Table S5: Bootstrapped internal validation of the PMRS predictors.
