# Supplementary material for: Development and Internal Validation of the Palliative Metabolic Risk Score (PMRS) for Predicting Critical Outcome in Palliative Inpatients
Source: Healthcare (Basel). 2026 Apr 15;14(8):1041. doi: 10.3390/healthcare14081041 (PMC13116770; doi:10.3390/healthcare14081041)
Supplement: Supplementary file 1 [file healthcare-14-01041-s001.zip › healthcare-4191968-supplementary.pdf]

Supplementary Table S1. Multiple linear regression for hospital length of stay

Variable	β Coefficient (Beta)	*p*-value
Pressure ulcer (present)	**+12.343**	**0.0020**
In-hospital mortality (death)	−7.062	0.0783
CRP (mg/L)	−0.071	0.0988
Age (years)	−0.079	0.5981
Mean glucose (mg/dL)	−0.017	0.5452
Number of comorbidities	+0.189	0.9359
Constant	30.311	0.0117

Note: Multiple linear regression showing predictors of hospital length of stay.
Pressure ulcer was the only significant predictor, prolonging stay by ≈12 days (p=0.002).
Negative coefficients indicate shorter length of stay.
CRP = C-reactive protein.

Supplementary Table S2. Univariate association between comorbidities and mortality

Comorbidity	Odds Ratio (OR)	95% Confidence Interval	*p*-value
Ischemic heart disease / heart failure	2.27	0.88 – 5.86	0.091
Stroke (SVO)	**0.17**	0.05 – 0.57	**0.004**
COPD	**0.18**	0.05 – 0.67	**0.011**
Dementia	0.47	0.22 – 1.03	0.060

Note: Univariate logistic regression assessing the association of major comorbidities with in-hospital mortality.
Bold indicates statistical significance (p<0.05).
COPD = chronic obstructive pulmonary disease; SVO = stroke.

Supplementary Table S3. Subgroup analysis in diabetic patients (n ≈ 61)

Variable	Odds Ratio (OR)	95% Confidence Interval	*p*-value
Oxygen requirement (present)	2.017	0.642 – 6.342	0.230
Mean glucose (mg/dL)	1.004	0.998 – 1.010	0.185

**Variable**	**Odds Ratio (OR)**	**95% Confidence Interval**	***p*-value**
CRP (mg/L)	1.006	0.995 – 1.017	0.288
Albumin (g/L)	0.975	0.928 – 1.024	0.289
Cancer as primary diagnosis	1.641	0.482 – 5.589	0.429
Age (years)	1.017	0.979 – 1.056	0.373

Note: Logistic regression limited to the diabetic subgroup.
None of the predictors reached statistical significance, likely due to low sample size and limited events per variable (EPV <10). CRP = C-reactive protein.

Supplementary Table S4. Bootstrapped internal validation of the PMRS predictors (1000 resamples)

Predictor	Original OR	Bias-Corrected OR	Bootstrap 95% CI (Lower)	Bootstrap 95% CI (Upper)
Oxygen requirement	4.429	5.033	2.087	15.956
Mean glucose (mg/dL)	1.011	1.011	1.005	1.019
Albumin <25 g/L	2.231	2.273	1.166	4.568
Primary diagnosis: cancer	2.447	2.590	1.197	6.865
CRP >64.1 mg/L	1.605	1.608	0.793	3.178
Intercept	0.109	0.098	0.030	0.265

Note: The bootstrap procedure yielded consistent effect estimates, confirming the robustness of the five predictors included in the PMRS model. Bias-corrected odds ratios remained close to the original values, and all key variables—particularly oxygen requirement, glucose level, albumin <25 g/L, and malignancy—retained statistical significance in the resampled datasets. These results support the internal stability of the model.

Supplementary Table S5.Exploratory multivariable logistic regression analyses evaluating the interaction between diabetes mellitus and glucose levels for prediction of critical outcome

Variable	OR	95% CI	p-value
**Model 1: Interaction model (DM × glucose)**			
Primary diagnosis (cancer)	0.381	0.169 – 0.856	**0.020**
Oxygen requirement	0.224	0.093 – 0.537	**0.001**
CRP >64.1 mg/L	0.477	0.251 – 0.904	**0.023**
**DM × glucose interaction**	**1.005**	**1.001 – 1.008**	**0.018**
**Model 2: Interaction model with centered glucose**			
Primary diagnosis (cancer)	0.396	0.178 – 0.883	**0.024**
Oxygen requirement	0.256	0.108 – 0.606	**0.002**
CRP >64.1 mg/L	0.494	0.262 – 0.931	**0.029**
**DM × centered glucose interaction**	**1.009**	**0.999 – 1.018**	**0.065**

Abbreviations: *DM: diabetes mellitus; CRP: C-reactive protein; OR: odds ratio; CI: confidence interval.*
***Note**: Centered glucose was calculated as fasting glucose minus the cohort mean (168.4 mg/dL) to reduce collinearity in the interaction model.*
